# Summary of the Experience in the Diagnosis and Treatment of Complex Preauricular Fistulas in 78 Children

**DOI:** 10.3389/fsurg.2020.609852

**Published:** 2021-02-01

**Authors:** Ying Xu, Dongjie Seng, Lan Jiang, Shengcai Wang, Xin Ni, Jie Zhang, Fugen Han

**Affiliations:** ^1^Department of Otorhinolaryngology Head and Neck Surgery Henan Children's Hospital, Department of Otorhinolaryngology Head and Neck Surgery, Zhengzhou Children's Hospital, Zhengzhou, China; ^2^Department of Otolaryngology Head and Neck Surgery, National Center for Children's Health, Beijing Children's Hospital, Capital Medical University Beijing, Beijing, China

**Keywords:** congenital preauricular fistula, surgical treatment, plastic surgery, mini-incision, children

## Abstract

**Objective:** This study aimed to investigate the application of mini-incisions in complex preauricular fistula resection in children.

**Methods:** A total of 78 children who were diagnosed with preauricular fistula infection between January 2017 and December 2019 were included in the study. Their clinical data were analyzed retrospectively, and surgical treatment with mini-incisions based on plastic surgery principles and techniques was provided.

**Results:** All the patients achieved healing following the first application of the treatment. The patients were followed up for 6–42 months, and no recurrence or local auricular deformation occurred.

**Conclusion:** The application of mini-incisions and plastic surgery techniques in complex preauricular fistula resection in children can achieve a good effect and improve the satisfaction of the children and their parents.

## Introduction

Congenital preauricular fistula (CPF) is a common congenital malformation of the external ear in children. In most patients, it manifests as a skin pit in front of the ascending branch of the helix. No intervention should be implemented when the patients have no obvious symptoms. However, if they present with red swelling, pain, and/or surrounding skin ulceration and other infection symptoms, the traditional treatment is incision and drainage, and surgical resection is performed after the inflammation is essentially controlled (1). Preauricular fistula infection usually begins to develop in infants and young children and requires incision and drainage, as well as frequent dressing changes. However, since infants and children cannot cooperate, parents are required to assist in the dressing changes, which can cause serious psychological harm to both the children and their parents. In the clinic, recurrent preauricular fistula or preauricular fistula with periauricular granuloma or scar formation caused by repeated infection after fistulectomy is called complex preauricular fistula (2). In such cases, the timing of the operation and incision design are important to achieve good results. The details of the present study are reported below.

## Data and Methods

### Clinical Data

A total of 78 children who were diagnosed with preauricular fistula infection at the Department of Otorhinolaryngology/Head and Neck Surgery at Children's Hospital Affiliated to Zhengzhou University between January 2017 and December 2019 were included in the study, and their clinical data were analyzed retrospectively. Among these patients, 32 were male and 46 were female. The youngest was 3 months old, and the oldest was 14 years old; the median age was 5.5 years. The fistula was in the left ear in 46 patients and in the right ear in 32 patients. All the patients had a history of repeated infection before admission, and 37 had a history of local abscess incision and drainage. All the patients with preauricular fistula infection were operated on under general anesthesia with endotracheal intubation by the same physician. The electrocardiography (ECG), chest X-ray imaging, routine blood test, anticoagulant function, and liver and kidney function tests were conducted before the operation, and the clinical data of all the children were collected and sorted.

### Surgical Methods

All the patients were anesthetized by tracheal intubation and intravenous anesthesia. The ear to be operated on was routinely disinfected, and surgical drapes were placed in position. The areas around the fistula were locally infiltrated with normal saline and injected with methylene blue solution for labeling. A small fusiform incision was made at the orifice of the fistula (the incision size was determined according to the child's age and was as parallel to the foot of the helix as possible), and the spontaneous rupture or drainage incision was retained (a small rubber drainage strip was placed in position after the operation). Microscopy was not used for our procedures. The skin and subcutaneous tissue were separated, and the surrounding tissues were incisively separated along the fistula or cyst to expose the perichondrium of the ear helix. The perichondrium of the ear was incised, and then the fistula or cyst was incised along the perichondrium to the apophysis helicis. In most patients, the fistulas or cysts adhered tightly to the cartilage. A small part of the adhered cartilage was removed. The separation range was deep, as far as the superficial layer of the temporal muscle fascia, and the front boundary reached the ulcerated skin or granulation scar tissue. The posterior boundary reached the deep layer of the posterior margin of the cartilage of the crus of the helix, and the lower boundary reached the crus of the helix. Attention was given to protect the integrity of the parotid gland capsule. For the rupture or drainage incision, the granulation tissue was scraped to the normal tissue with a curette. After hemostasis, the operative cavity was washed with normal saline, and the incision was sutured in alignment. To avoid invagination of the incision edge, vertical mattress suturing was performed in the center. The rupture or drainage incision was sutured with reduced tension, and a small drainage strip was placed in position. The external auditory canal, triangular fossa, scaphoid fossa, and auricular concha cavity were filled with gauze, and a compression bandage was applied for 2 days. After 2 days, the drainage strip was removed. Broad-spectrum antibiotics were administered intravenously or applied according to a drug sensitivity test for 3 days. The stitch removal was conducted 7–10 days after the operation.

## Results

**Figure 1:**
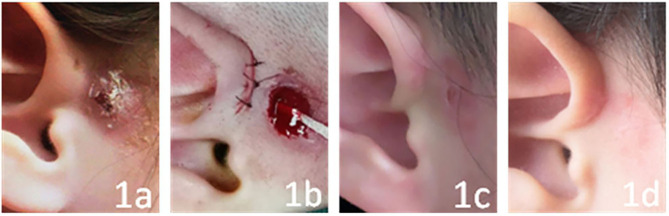
The child has had fistula infection for 6 months, and it is chronic. **(a–d)** show the conditions before the operation, just after the operation, and at 1 week and 3 months after the operation, respectively.

**Figure 2:**
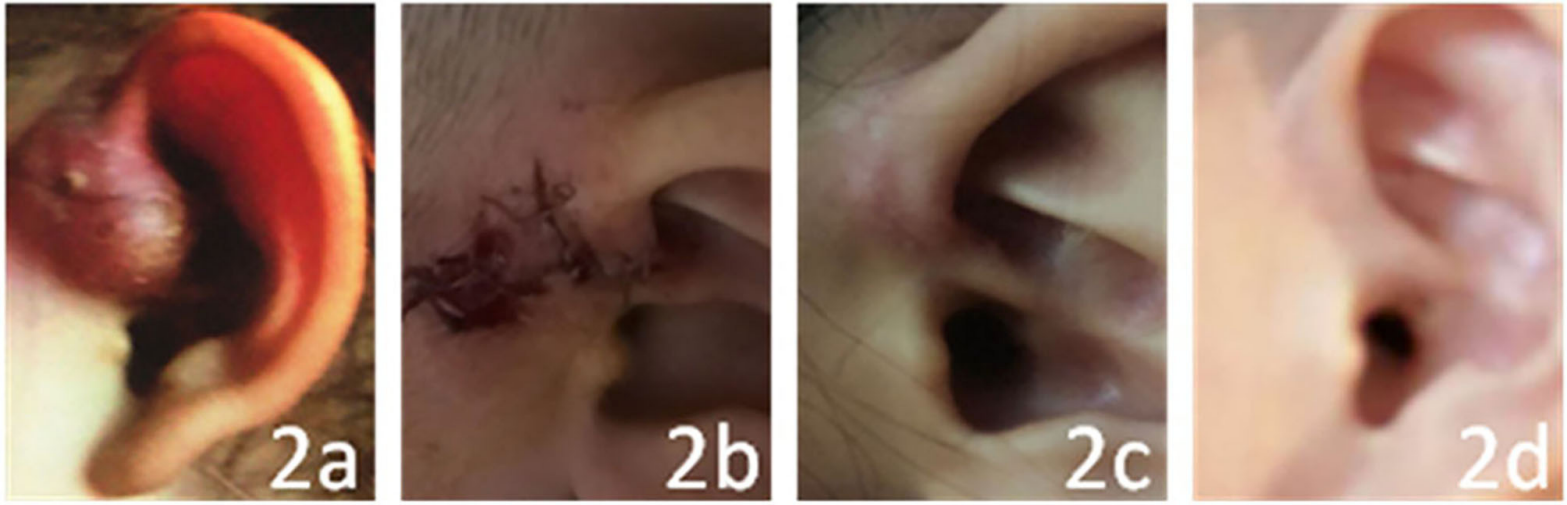
The child has abscess formation after incision and drainage. **(a–d)** show the conditions before the operation, just after the operation, and at 1 week and 3 years after the operation, respectively.

In this study, the operation on all the children was successful, and the effect was satisfactory. Only 1 of the 78 children had a dead space induced by the operation. Hematoma formation and secondary infection occurred after drainage on the second day after the operation. The child was treated with intravenous broad-spectrum antibiotics for 3 days, the area was debrided and sutured, and a compression bandage was applied. The area healed after 1 week. The incisions of the other 77 children were concealed and healed in the first stage, and no auricle malformation was found. The patients were followed up for between 6 months and 3 years, and no recurrence occurred (see Figures 1, 2 for two specific children).

## Discussion

Preauricular fistula is a congenital external ear disease with many branches and blind tubes and is divided into three types: simple, infective, and secretory. A simple fistula does not need treatment; surgical treatment is required only when the fistula secretes for a long time or an abscess is formed by infection. In addition to the classic shuttle incision around the fistula and resection along with fistula tracing to the blind end, other surgical methods include local block resection (3), extended resection (4), microscopic resection (5), and double incision combined with Z-plasty (6). A previous study reported that the postoperative recurrence rate is 0–43%.

For complex preauricular fistula in children with repeated infection, parents have high expectations of surgery. This requires doctors to consider not only completely eradicating the lesions, but also the trauma of the surgery itself, wound repair after resection, and scar constitution of the children to avoid auricle deformation and reduce scar formation and related cosmetic problems.

### Timing of Operation

For patients with acute preauricular fistula infection, the traditional treatment concept is that those with an abscess should be systematically treated with antibiotics. Incision and drainage are performed first, and then the surgery is performed after the infection is controlled. Choosing the correct timing of the operation is important to reduce the pain of the children and their parents, and to reduce the recurrence rate after the operation. Zheng stated that as long as the local edema is alleviated and the skin color changes from an obviously congested red to a dark red, this means that the infection has been controlled (7). This is the best timing for an operation for CPF with infection or abscess; there is no need to incise the abscess since scar tissue has not yet formed. Under direct observation, it is easier to completely remove the necrotic material and reduce the recurrence. Therefore, it is of great clinical significance to perform preauricular fistulectomy in the infection stage during the treatment of CPF. It was revealed in this study that the timing of the operation should observe the following principles:

(1) If a preauricular fistula infection abscess does not form, the preoperative examination should be completed, and the operation should be performed.
(2) Patients with an acute preauricular fistula infection abscess should be treated with local incision and drainage. The pus cavity should be filled with gauze after the pus is removed, and a dressing change should be carried out every day. In addition, sensitive antibiotics or empirically used antibiotics sensitive to common pathogenic bacteria such as *Staphylococcus, Streptococcus*, and *Proteus* should be selected for treatment according to the bacterial culture and drug sensitivity test results. If there is no fluid leakage from the fistula irrigation and the distance between the wound edge and the skin of the fistula is over 3 mm, surgery should be performed.
(3) In the case of a preauricular fistula infection abscess developing into chronic inflammation, if the infected skin or the distance between the incision and the fistula is over 3 mm, an operation should be performed. It was found that the majority of this type is secretory preauricular fistula, and that the cyst adheres to the perichondrium of the ear helix.

### Surgical Methods

The classic preauricular fistulectomy method is to make a shuttle incision around the fistula after injection of methylene blue, and then remove the fistula along with the blind end (8–10). Zhou et al. revealed that for preauricular fistulectomy with the helix spine as the anatomical mark, the recurrence rate is 4.0% (11). However, the recurrence rate could be reduced to 1.9% after using microscopy (5).

In this study, 78 children with preauricular fistula infection were selected. In all the children, a small spindle incision was made along the fistula, and then the skin and subcutaneous tissue were incised to expose the perichondrium of the ear helix and separated along the perichondrium to expose the fistula and cyst. Almost all the secretory and infected preauricular fistulas were adhered to the perichondrium of the ear helix. In these cases, part of the cartilage had to be removed, and damage to the cartilage of the helix angle was avoided as much as possible. In this study, the longest follow-up period was 3 years, and no recurrence was found. One child had a dead space due to suturing; hematoma formation and secondary infection occurred after drainage on the second day after operation, but the child healed after the area was debrided and sutured and a compression bandage was applied. This also indicated that the operative cavity is larger after the fistula tissue and granulation tissue are excised, especially in secretory preauricular fistulas with infection. It is difficult to eliminate a dead cavity by suturing layer by layer, but intermittent sutures should be used. The middle part is sutured vertically to prevent a varus. After the operation, the external auditory canal, concha cavity, scaphoid fossa, and triangular fossa are filled with gauze, and then compression bandages are applied. If there is any defect after the granulation tissue is removed from the wound or scar in front of the fistula, the subcutaneous tissues are separated and sutured with reduced tension, and small drainage strips are placed in position.

### Width and Depth of Resection

Anatomically, when a fistula is blocked, it expands into a cyst due to accumulation of local secretions, with the upper boundary at the ear cranial junction, the lower boundary at the upper edge of the parotid gland, the front boundary at the edge of growth, and the posterior border at the posterior of the deep surface of the perichondrium of the ear helix, i.e., it extends from the basement to superficial layers of the temporal muscle fascia (12). Generally, fistulas and granulation tissues do not cross the superficial layer of the temporal muscle fascia. They also have safety boundaries with the parotid gland and facial nerve and are separated along the perichondrium of the ear helix during resection to expose the cartilage of the helix. Then, the lesions and granulation tissues are dissected along the superficial layer of the temporal muscle fascia downward toward the helix angle to excise the helix spine and part of the cartilage and backward toward the posterior deep surface of the perichondrium of the ear helix. During resection, the capsule and the granulation tissue outside the capsule are used as boundaries. In the infection stage, the fistula and inflammatory tissue should be removed together to achieve healing with the first application of the treatment.

### Local Repair

When the preauricular fistula becomes repeatedly swollen (purulent or local), this indicates that the fistula is secretory. If the lesion has chronic inflammatory granulation tissue and a bean curd residue-like substance that adheres closely to the cartilage, it will be difficult to heal the patient by treating with a simple anti-infection protocol and local dressing changes. Moreover, it will be difficult to assess the psychological trauma to the children and their parents (12–14). Guo reported that plastic and cosmetic surgery play important roles in complex preauricular fistula resection and repair (15). During the operation, according to the relationship between the infection focus and fistula, different incisions are designed, and skin flaps are made to repair the wound; thereby, good surgical results can be achieved. Although preauricular fistulas generally do not require treatment (16), fistulizing infections, if not properly treated, can lead to recurrent infections and result in significant postoperative scarring (17). Scheinfel et al. also considered surgical resection important for recurrent preauricular sinus infection (18). In addition, postoperative management is critical, and the study conducted by Tian et al. found that if a patient develops an abscess, open drainage should be considered, and the specimen sent for bacterial culture (19). In this study, a small incision was made along the fistula to ensure a good cosmetic effect, and to protect the blood supply of the flap, sharp separation was performed during the operation. During the flap-making, the subcutaneous lesions had to be removed and the dermis of the flap preserved as much as possible. Furthermore, the skin had to be sutured without tension.

In summary, the complex preauricular fistula operation in children is safe and effective. It can shorten the treatment time and reduce the pain of the children and their parents. Moreover, the postoperative recovery is good, and the recurrence rate is low. However, the operation in the infection stage has higher requirements for the surgeon. It is recommended that when performing such an operation, the surgeon should master the embryonic development, pathology, and anatomy of the preauricular fistula to ensure a good effect of the operation. A limitation of this study is that it only summarizes the experience of children with complex preauricular fistulas in terms of the timing of surgery, surgical approach, and breadth and depth of resection, and we did not discuss the difference between our technique and that of others.

## Data Availability Statement

The original contributions presented in the study are included in the article/supplementary material, further inquiries can be directed to the corresponding author/s.

## Ethics Statement

The studies involving human participants were reviewed and approved by Ethics Committee of Henan Children's Hospital. Written informed consent to participate in this study was provided by the participants' legal guardian/next of kin. Written informed consent was obtained from the individual(s), and minor(s)' legal guardian/next of kin, for the publication of any potentially identifiable images or data included in this article.

## Author Contributions

YX and FH conceived the idea and conceptualized the study and drafted the manuscript. DS and SW collected the data. LJ and XN analyzed the data. JZ reviewed the manuscript. All authors contributed to the article and approved the submitted version.

## Conflict of Interest

The authors declare that the research was conducted in the absence of any commercial or financial relationships that could be construed as a potential conflict of interest.

## References

[1] Chen B Jin T Wang C Wang R Ma H Zhang S Curative effect of surgical resection of preauricular fistula complicated with infection and abscess. Chin J Otorhinolaryngol Skull Base Surg. (2019) 25:297–9. 10.11798/j.issn.1007-1520.201903016

[2] Hu W Huang J Xue Z Mao L Jiang Y Zhen X Radical resection of recurrent preauricular fistula with infection. Chin J Otol. (2015) 4:761–3.

[3] Yao D Li C Cai W Regional preauricular continuous block resection for treating infective preauricular sinus of children. J Mol Imag. (2019) 42:551–3. 10.12122/j.issn.1674-4500.2019.04.30

[4] Zhang D Qin G Zhao C Zhu L Liu Y Li L. En Bloc pre-auricular tissue excision for treatment of refractory pre-auricular fistula. Chin J Otol. (2014) 2:304–6. 10.3969/j.issn.1672-2922.2014.02.30 29937802 PMC6002584

[5] Wang C Analysis of the curative effect of surgical treatment of infected ear anterior fistula. China For Med Treat. (2019) 38:85–7. 10.16662/j.cnki.1674-0742.2019.13.085

[6] Zhen L Li D Zeng Z Wu B Treatment for skin and auricular cartilage in lesion area by infection stage surgery for congenital preauricular fistula. China Pract Med. (2014) 20:263–4.

[7] Zheng S. The timing and techniques of congenital preauricular fistula infection during operation. J Clin Otorhinolaryngol Head Neck Surg. (2016) 30:1557–8. 10.13201/j.issn.1001-1781.2016.19.013 29871140

[8] Yoo H Park DH Lee IJ Park MC. A surgical technique for congenital preauricular sinus. Arch Craniofac Surg. (2015) 16:63–6. 10.7181/acfs.2015.16.2.63 28913224 PMC5556851

[9] Zhou P Chen J Huang T Tao J. Research progress of congenital preauricular fistula. J Clin Otolaryngology Head Neck Surg. (2019) 33:474–7. 10.13201/j.issn.1001-1781.2019.05.024 31163564

[10] Lee KH Lee SM Kim SW Park KJ Lee JH. Minimization of skin incision at preauricular sinusectomy using a trans pit approach. Int J Pediatr Otorhinolaryngol. (2020) 132:109903. 10.1016/j.ijporl.2020.109903 32014737

[11] Zhou P Chen J Huang T Tao J Analysis of curative effect and related factors of recurrence of preauricular fistula excision with spine of helix as anatomic marker. Chin Arch Otolaryngol Head Neck Surg. (2019) 26:194–7. 10.16066/j.1672-7002.2019.04.006

[12] Yin DP Dou XW Zhang HG Zhu HE Fan MY. Application experiences of local flap in the resection of the children's infectious congenital preauricular fistula. J Clin Otorhinolaryngol Head Neck Surg. (2016) 30:1968–9. 10.13201/j.issn.1001-1781.2016.24.018 29798278

[13] Wang YF Su JZ Song YL Cui L Geng JQ Zhao HT Analysis of misdiagnosis and surgical treatment of 16 cases in children with congenital preauricular fistula complicated with retroauricular infection. J Clin Otorhinolaryngol Head Neck Surg. (2017) 31:388–9. 10.13201/j.issn.1001-1781.2017.05.014 29871268

[14] Liu Z Cui J Application of rhomboid skin flap in wound repair after resection of preauricular fistula with infection. Chin J Anat Clin Pract. (2014) 19:332–3. 10.3760/cma.j.issn.2095-7041.2014.04.019

[15] Guo Y Application of plastic surgery techniques in complex congenital preauricular fistula. Fudan Univ J Med Sci. (2014) 41:647–50. 10.3969/j.issn.1672-8467.2014.05.013

[16] Gur E Yeung A Al-Azzawi M Thomson H. The excised preauricular sinus in 14 years of experience: is there a problem? Plast Reconstr Surg. (1998) 102:1405–8. 10.1097/00006534-199810000-00012 9773994

[17] Tan T Constantinides H Mitchell TE. The preauricular sinus: a review of its aetiology, clinical presentation and management. Int J Pediatr Otorhinolaryngol. (2005) 69:1469–74. 10.1016/j.ijporl.2005.07.008 16125253

[18] Scheinfeld NS Silverberg NB Weinberg JM Nozad V. The preauricular sinus: a review of its clinical presentation, treatment, and associations. Pediatr Dermatol. (2004) 21:191–6. 10.1111/j.0736-8046.2004.21301.x 15165194

[19] Tian H Zhong C. Postoperation of preauricular fistula cellulitis caused by methicillin-resistant staphylococcus aureus infection. J Otol. (2018) 13:111–3. 10.1016/j.joto.2018.07.002 30559776 PMC6291634

